# Auditory Function in the Tc1 Mouse Model of Down Syndrome Suggests a Limited Region of Human Chromosome 21 Involved in Otitis Media

**DOI:** 10.1371/journal.pone.0031433

**Published:** 2012-02-14

**Authors:** Stephanie Kuhn, Neil Ingham, Selina Pearson, Susan M. Gribble, Stephen Clayton, Karen P. Steel, Walter Marcotti

**Affiliations:** 1 Department of Biomedical Science, University of Sheffield, Sheffield, United Kingdom; 2 Wellcome Trust Sanger Institute, Wellcome Trust Genome Campus, Hinxton, Cambridge, United Kingdom; Kaohsiung Chang Gung Memorial Hospital, Taiwan

## Abstract

Down syndrome is one of the most common congenital disorders leading to a wide range of health problems in humans, including frequent otitis media. The Tc1 mouse carries a significant part of human chromosome 21 (Hsa21) in addition to the full set of mouse chromosomes and shares many phenotypes observed in humans affected by Down syndrome with trisomy of chromosome 21. However, it is unknown whether Tc1 mice exhibit a hearing phenotype and might thus represent a good model for understanding the hearing loss that is common in Down syndrome. In this study we carried out a structural and functional assessment of hearing in Tc1 mice. Auditory brainstem response (ABR) measurements in Tc1 mice showed normal thresholds compared to littermate controls and ABR waveform latencies and amplitudes were equivalent to controls. The gross anatomy of the middle and inner ears was also similar between Tc1 and control mice. The physiological properties of cochlear sensory receptors (inner and outer hair cells: IHCs and OHCs) were investigated using single-cell patch clamp recordings from the acutely dissected cochleae. Adult Tc1 IHCs exhibited normal resting membrane potentials and expressed all K^+^ currents characteristic of control hair cells. However, the size of the large conductance (BK) Ca^2+^ activated K^+^ current (*I*_K,f_), which enables rapid voltage responses essential for accurate sound encoding, was increased in Tc1 IHCs. All physiological properties investigated in OHCs were indistinguishable between the two genotypes. The normal functional hearing and the gross structural anatomy of the middle and inner ears in the Tc1 mouse contrast to that observed in the Ts65Dn model of Down syndrome which shows otitis media. Genes that are trisomic in Ts65Dn but disomic in Tc1 may predispose to otitis media when an additional copy is active.

## Introduction

Down Syndrome, a chromosomal disorder caused by the presence of an additional copy (complete or part: trisomy) of chromosome 21, Hsa21, is characterised by some impairment of cognitive ability and physical growth and a particular set of facial characteristics amongst other things [1], [2]. About 1 in every 700 newborns is affected with Down Syndrome [1]. One feature often seen and of particular interest to this study, is the relatively high incidence of otitis media (in up to 78% of patients) and conductive hearing impairment in people with Down Syndrome. However, with correct diagnosis and treatment up to 98% of patients have normal hearing [3]. Several mouse models of Down syndrome have been developed [2]. Recently, one of these mouse models for Down Syndrome has been reported exhibiting otitis media (Ts65Dn mice). These mice demonstrated high but variable ABR thresholds, many had middle ear effusion and often showed a thickened middle ear mucosa compared to wildtype controls [4].

In the present study we have examined another mouse model of Down syndrome (Tc1), which carries most of human chromosome 21 (Hsa21) and shows many phenotypic similarities to Down syndrome [5]–[7], to look for signs of hearing impairment, otitis media and possible defects of the cochlear sensory hair cells. Here we show that despite the increased expression of the K^+^ current (*I*_K,f_) characteristic of mature IHCs, the middle and inner ear structure and functional hearing were indistinguishable between control and Tc1 mice. As only parts of human chromosome 21 have been found to be included in the Tc1 mouse, the predisposition to otitis media in Down syndrome may be associated with genes that are not functionally trisomic in the Hsa21 chromosomal material in Tc1 mice.

## Results

### Sensitivity of hearing in Tc1 mice

**Figure 1:**
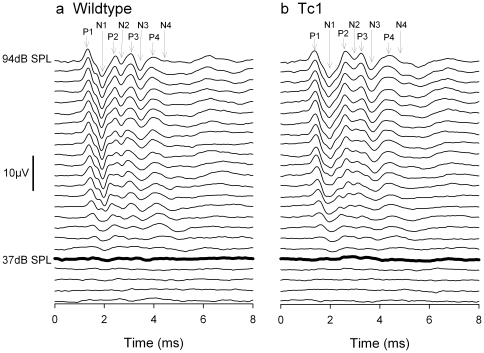
Click-evoked ABR waveforms in control and Tc1 mice. Averaged ABR waveforms recorded in response to clicks in a wildtype (a) and Tc1 (b) mouse. Waveforms are displayed for stimuli in 3 dB incremental steps from 25 dB SPL at the bottom to 94 dB SPL at the top of each panel. Positive peaks 1–4 (P1–P4) and negative peaks (N1–N4) are indicated by arrows at the top of each panel. The heavy black line indicates the visually-determined threshold; 37 dB SPL in both cases. The scale-bar indicates 10 µV amplitude.

**Figure 2 pone-0031433-g002:**
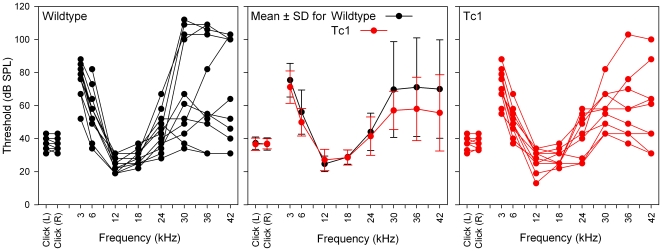
ABR Thresholds in control and Tc1 mice. ABR thresholds for individual control (black circles) and Tc1 (red circles) mice are shown on the left and right panels respectively. The middle panel indicates the mean (± standard deviation, SD) thresholds for control (black) & Tc1 (red) mice, respectively. Tone-evoked ABRs were recorded over the left side of the head only whereas click-evoked ABRs were recorded bilaterally.

ABRs were recorded and used to determine the threshold of hearing for click and tone pip stimuli in wildtype control (11.8±0.4 weeks, *n* = 11, 7 males, 4 females) and Tc1 mice (11.8±0.4 weeks, *n* = 10, 4 males, 6 females). Examples of click-evoked ABR waveforms are shown in Figure 1. There were no obvious or systematic differences in waveforms recorded from the two groups. In all mice, the mean ABR threshold audiograms showed a characteristically normal profile, having a region of highest sensitivity (lowest threshold) in the 12–18 kHz region (Fig. 2). Frequencies above and below this region had typically higher thresholds. In both Tc1 and control groups, thresholds at high frequencies showed considerable variation amongst individuals (Fig. 2A, 2C) which may be associated with the mixed genetic background that includes C57BL/6J and 129S8 and the fact that many mouse strains show progressive hearing loss [8]. Analysis of variance (Two-Way) indicated there were no significant differences between the thresholds of control and Tc1 mice (F = 0.781, p = 0.634). Click ABR thresholds were estimated by taking recordings from both the left and right sides of the head. There were no significant asymmetries in click ABR thresholds in either the control or Tc1 mice (Fig. 2) and no significant effect of the addition of human chromosomal material (Two Way ANOVA; F = 0.131, p = 0.719).

### Input-Output Functions

**Figure 3:**
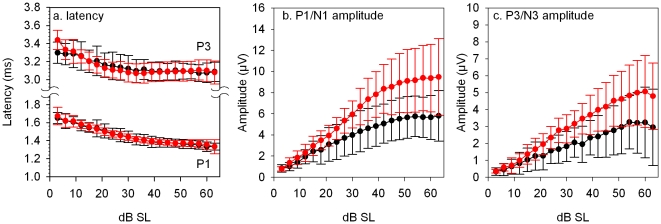
Input-Output Functions of Wave Latency. **A**, The mean latency (± SD) of 2 positive peaks in the ABR waveform (P1 and P3) are plotted as a function of dB SL (sensation level) for wildtype (black lines) and Tc1 (red lines) mice. B, **C**. The mean amplitude (± SD) of 2 positive-negative (P1-N1 and P3-N3) peaks in the ABR waveform are plotted as a function of dB SL for wildtype (black lines) and Tc1 (red lines) mice.

From click-evoked ABR waveforms, four positive and negative peaks could be discriminated. In Figure 3A, we plotted mean latency of positive peaks 1 and 3 (P1 and P3) as a function of dB SL (sensation level; i.e. dB above threshold) for wildtype and Tc1 mice. The mean positive-negative amplitude of waves 1 and 3 (P1/N1 and P3/N3) were plotted as a function of dB SL in wildtype and Tc1 mice (Fig. 3B–C, respectively). On the whole, there was no systematic effect of addition of extra chromosomal material on these waveform latency and amplitude parameters; the input-output function curves obtained from the mice were largely overlapping.

### Middle and inner ear appearance in Tc1 mice

**Figure 4:**
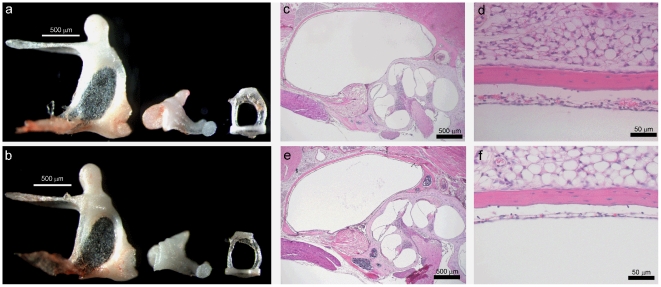
Middle Ears of Control and Tc1 mice. **A** and B, illustrate the malleus (left), incus (middle) and stapes (right), from a wildtype mouse and a Tc1 mouse, respectively, showing no differences. **C–F** illustrate sections through the mucosal lining of the middle ear (H&E stained) for a wildtype (**C**, **E**) and Tc1 (**D**, **F**) mouse, showing no sign of any middle ear inflammation.

**Figure 5:**
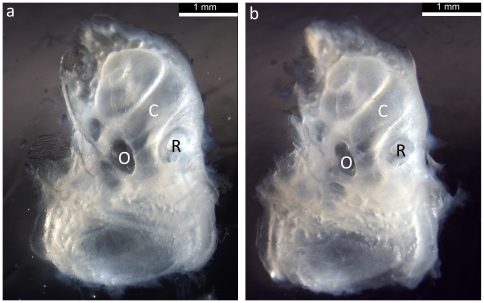
Cleared Inner Ears from Control and Tc1 mice. Gross morphology of the inner ears of a control and Tc1 mouse are shown in **A** and **B**, respectively. The cochlear duct (C) is visible as a spiral structure towards the top of each image. The oval (O) and round (R) windows can be seen in the middle of each panel. The semicircular canals of each inner ear are found in the wider region at the bottom of each image. No abnormalities can be detected in Tc1 inner ears.

Following ABR recordings, the middle ear was carefully dissected and examined for signs of any inflammation or malformation. Minor anomalies were detected in some individuals such as a small amount of fluid in the middle ear, slight whitening of the bone surrounding the middle ear cavity, or some cerumen accumulating in the external ear canal, but these features were all detected in both Tc1 and control mice and were not correlated with raised thresholds observed at high frequencies. Ossicles showed a normal appearance in Tc1 mice (Fig. 4A, 4B). Sections of the middle ears revealed no evidence of effusion in the middle ear cavities and no signs of thickening of the mucosa lining the middle ear of Tc1 or control mice (Fig. 4C–F). Examples of cleared inner ears from mice of both groups are shown in Figure 5. No gross structural abnormalities were detected in the inner ears of control or Tc1 mice.

### The expression of adult-type ion channels in Tc1 mutant hair cells is normal

Sound detection depends on the sensory hair cells present in the cochlea. The role of hair cells is to transduce mechanical acoustic energy into an electrical signal [9]. While IHCs, the primary sensory receptors of the mammalian cochlea, are responsible for relaying sound information to the central nervous system via auditory nerve fibres [10], OHCs act in parallel to enhance the sensitivity and frequency selectivity of the cochlea by active mechanical amplification [11]–[13].

**Figure 6:**
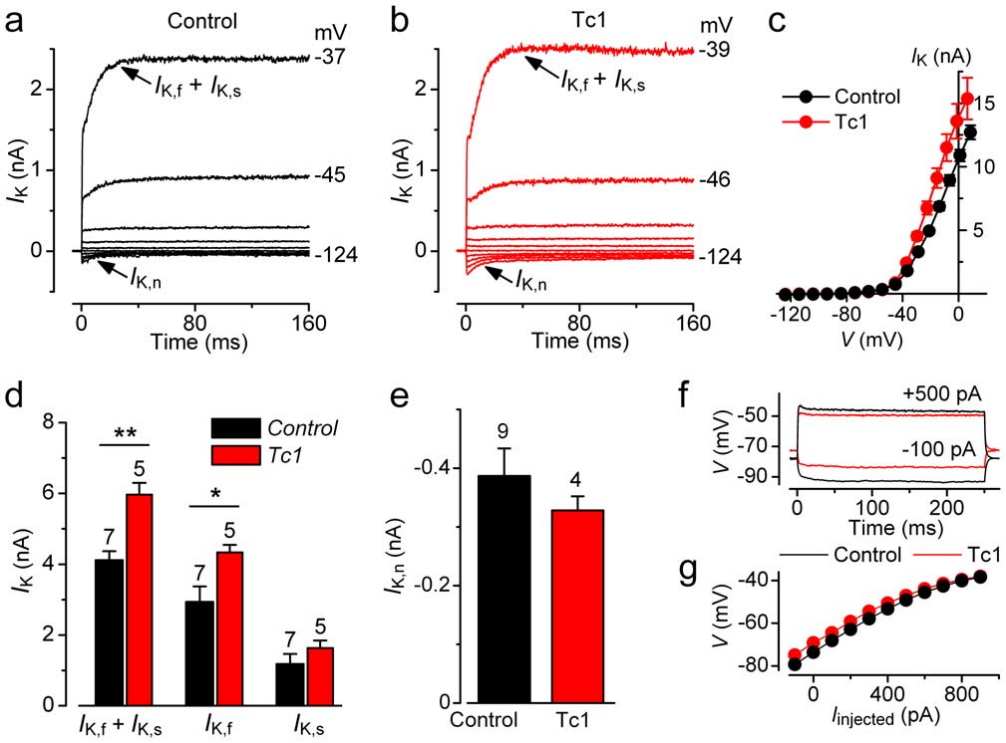
Current and voltage recordings in IHCs from adult Tc1 mice. **A** and **B**, Membrane currents recorded from adult (P25) control and Tc1 apical coil IHCs. Currents were elicited by depolarizing voltage steps in 10 mV nominal increments from −124 mV to the various test potentials shown by some of the traces (holding potential −84 mV). **C**, Average steady-state current-voltage (*I*–*V*) curves obtained from control (P25–P26, *n* = 7) and Tc1 (P25–P26, *n* = 5) adult IHCs. **D**, Average total K^+^ current (*I*_K,f_+*I*_K,s_) and isolated *I*_K,f_ and *I*_K,s_ measured at the membrane potential of −25 mV. E, Size of *I*_K,n_ in control and Tc1 mutant IHCs. **F**, Voltage responses under current clamp from control and Tc1 adult IHCs (P25). Current steps were applied between −100 pA and +900 pA, starting from the resting potential, and for clarity only a few responses are shown. **G**, non-linear behaviour of the peak voltage responses of IHCs, obtained from the protocol described in panel **F**. In this and the following Figure, values are shown as mean ± SEM. Recordings are at body temperature.

**Figure 7:**
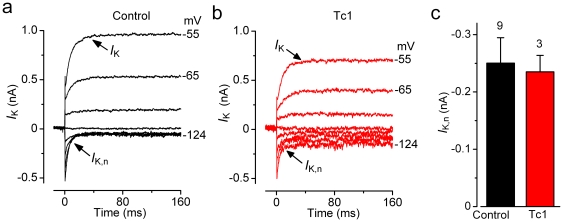
Current recording in OHCs from adult Tc1 mice. **A** and B, Typical current responses from apical-coil adult control (P14) and Tc1 (P13) OHCs. Currents were elicited by depolarizing voltage steps (10 mV nominal increments) from −124 mV (holding potential of −84 mV). **C**, Steady-state I–V curves for control (P13–P14, *n* = 9) and Tc1 (P13–14, *n* = 3) OHCs. **D**, Average size of the total K^+^ current at the steady-state (*I*_K_+*I*_K,n_) and the isolated I_K,n_.

We investigated the biophysical properties of adult inner (IHCs) and outer (OHCs) hair cells from Tc1 mice using patch clamp recordings, to test whether additional copy (complete or part) of chromosome 21 (trisomy 21) had any effect on the normal function or development of these cells. Both control and Tc1 IHCs from adult mice exhibited a large K^+^ current (Fig. 6A–C). However, the total steady-state outward K^+^ current, measured at −25 mV, was found to be significantly larger in Tc1 than that in littermate control IHCs (p<0.005: Fig. 6D). When we tested which of the K^+^ current characteristics of adult IHCs (*I*_K,f_ and *I*_K,s_ [14] and *I*_K,n_ [15]) was affected by the additional human DNA, we found that only *I*_K,f_, a large conductance Ca^2+^-activated K^+^ current, was up-regulated in Tc1 IHCs (Fig. 6D, 6E). Despite the larger amplitude the activation of *I*_K,f_, defined as time to reach half-maximal activation [15], was found to be not significantly different between Tc1 (0.30±0.03 ms, n = 5) and littermate control (0.28±0.04 ms, n = 7) IHCs, suggesting that the additional human DNA is only affecting the expression level and not the intrinsic properties of the channel. We then investigated whether a larger *I*_K,f_ in mutant IHCs had an effect on the cell's voltage responses. In both control and mutant IHCs, hyperpolarizing and depolarizing current injections from the resting membrane potential, elicited fast, graded voltage responses (Fig. 6F) as previously observed [14], [16]. In Tc1 IHCs, current steps appeared to cause significantly smaller voltage responses, which was more evident from the relation between injected current and peak voltage responses (p<0.0001, Fig. 6G). Although the increased *I*_K,f_ affected the receptor potential, the general biophysical properties of Tc1 IHCs (such as resting membrane potentials and linear leak conductance) did not differ from those of control cells (see Table 1). Similar to IHCs, the K^+^ current characteristic of adult OHCs, *I*_K,n_ [17], was also equally expressed in both control and Tc1 OHCs (Fig. 7).

**Table 1:** Electrical properties of IHCs (P25–P26) and OHCs (P13–P14) from control and Tc1 mice.

	Control	Tc1
**IHCs (P25–P26)**		
*C*_m_, pF	9.9±0.3 (9)	10.5±0.4 (5)
*V*_m_, mV	−73.7±1.2 (9)	−69.8±1.2 (5)
g_Leak_, nS	1.0±0.2 (9)	1.6±0.4 (5)
*V*_Rev_, mV	−59.7±2.62 (7)	−59.7±4.06 (3)
**OHC (P13–P14)**		
*C*_m_, pF	8.67±0.31 (9)	7.33±0.48 (3)
g_Leak_, nS	3.07±0.25 (9)	2.60±0.10 (3)

Values are means ± SEM; number of cells is in parentheses. *C*_m_ = membrane capacitance; *V*_m_ = resting membrane potential; g_Leak_ = resting leak conductance; *V*_Rev_ = reversal potential of the total current.

## Discussion

Prior to the onset of hearing (postnatal day 12 in most rodents [18]) immature hair cells have to follow a developmental program that consists of the acquisition and/or elimination of different ion channels and synaptic proteins [15], [19]–[21]. One of these channels, which carries the large conductance K^+^ current *I*_K,f_, is characteristic of mature IHCs and enables them to operate fast voltage responses essential for accurate sound encoding. We found that *I*_K,f_ was slightly up-regulated in IHCs from Tc1 (Fig. 6) mice carrying part of human chromosome 21 (Hsa21), involved in Down syndrome in humans [5]. The gene encoding BK channels, *KCNMA1*, is not located on Hsa21 [22], [23], suggesting that the up-regulation of *I*_K,f_ is a secondary effect caused by activation or modulation of other intracellular signals in Tc1 IHCs. There was no apparent effect of *I*_K,f_ upregulation on hearing sensitivity, as determined by ABR threshold measurements (Fig. 2).

Recently, it has been shown that five miRNA genes are overexpressed in the heart and brain of people with Down syndrome [24], three of which (miR-99a, let-7c, miR-125b-2) are expressed in the inner ear [25]. MiRNAs are small non-coding RNAs able to regulate a broad range of genes involved in development, differentiation and human diseases [26], [27], including hearing [28]–[30]. However, the physiological functions for the majority of the known miRNAs are still not understood in most systems. Therefore, one possibility is that overexpression of a human chromosome 21-derived miRNA may exert some degree of gene regulation in cochlear IHCs, affecting BK channel expression. *Mirlet7c-1* (let-7c) is one of the genes on human chromosome 21 that is intact in the Tc1 mouse so may be overexpressed. Upregulation of miR-let-7c is expected to downregulate miR-17 via downregulation of c-myc [31]; miR-17 in turn is predicted to target Nr4a2 (Targetscan prediction; http://www.targetscan.org/) so downregulation of miR-17 should upregulate Nr4a2; finally, upregulation of Nr4a2 should lead to upregulation of Kcnma1 [32] which could explain the larger K^+^ current *I*_K,f_ in Tc1 IHCs.

Hearing sensitivity of Tc1 mice was comparable to wildtype controls (Figs. 1–3). There were no consistent changes in ABR waveform, amplitude or latency as a function of sound pressure level; a further indication of normal hearing in the Tc1 mice. Some minor anomalies were noted in the middle ears and ossicles of some mice (Fig. 4), but these did not correlate with any functional changes. Gross anatomy of the inner ears, as revealed by clearing of the tissues (Fig. 5), was also normal in both control and Tc1 mice. Thus, we concluded that addition of the majority of human chromosome 21 into the mouse genome does not lead to the otitis media phenotype seen in the Ts65Dn mouse model of Down Syndrome [4] and in Down Syndrome patients [33].

The Ts65Dn mouse model contains just under 60% of the mouse orthologues of the human genes on Hsa21 and produces behavioural and cognitive phenotypes similar to Down Syndrome as well as otitis media [4], [34], [35]. However, several genes known to regulate neuronal function are not triplicated in the Ts65Dn model (eg *S100b* and *Trpm2*) [36], [37]. Addition of Hsa21 into mice may also not produce a complete phenotype, for example due to potential differences in expression of human genes in a mouse cell. In the Tc1 line, the extra human DNA is now known to be more disrupted than was originally thought and may lead to inactivation of additional human genes on Hsa21 and consequently lack of trisomy (unpublished data). We carried out a detailed comparison of the genes demonstrating effective trisomy in the Ts65Dn and Tc1 mouse models to investigate which genes might be involved in the susceptibility to otitis media, as Ts65Dn mice show otitis media but Tc1 mice do not. Details of triplicated genes in Ts65Dn mice [38] were compared with the list of intact Hsa21 genes in Tc1 mice (unpublished data). Hsa21 orthologues that are trisomic in Ts65Dn but only disomic in Tc1 might predispose to otitis media when overexpressed, and these are listed in Table 2. Some of these genes appear to be involved in immune function because mouse knockouts show immune defects, including *Ifnar1*, *Ifnar2*, *Il10rb*, *Ifngr2*, *Kcne1*, *Rcan1* and *Runx1*, and these may underlie abnormal (overactive) immune responses to environmental triggers and hence susceptibility to otitis media when overexpressed instead.

**Table 2:** Genes trisomic in Ts65Dn but disomic in Tc1.

Name	Start (HG19)	End (HG19)	Human gene	Status of human gene in Tc1	Mouse gene	Mouse mutant reported?	Phenotype of mouse mutant (from MGI)	Candidate for otitis media?
NM_201413	27252860	27543138	APP	rearranged, non functional	*App*	Yes	Mice homozygous for disruptions in this gene exhibit reduced body weight, brain weight, size of forebrain commissures, locomotor activity, forelimb grip strength, spatial learning scores. Many mice also exhibit agenesis of the corpus callosum and extensive reactive gliosis.	
NM_018944	33640529	33651376	C21orf45/MIS18A	deleted	*2610039C10Rik*	No		
NM_206898	33664123	33687094	MRAP	deleted	*Mrap*	No		
NM_014825	33683329	33765312	URB1	deleted	*Urb1*	No		
NR_002996	33749495	33749631	SNORA80	deleted	Not found	Not found		
NR_026845	33765441	33766266	C21orf119	deleted	Not found	Not found		
NM_058187	33784751	33887697	C21orf63	deleted	*4931408A02Rik*	No		
NM_144659	33947150	33957845	TCP10L	deleted	*Tcp10a*	No		
NM_021254	33973983	33984918	C21orf59	deleted	*1110004E09Rik*	No		
NM_003895	34001068	34100351	SYNJ1	deleted	*Synj1*	Yes	Homozygotes for a targeted null mutation exhibit neurological defects associated with impaired phosphoinositide metabolism and accumulation of clathrin-coated vesicles at nerve endings. Mutants show impaired suckling and most die within 24 hours of birth.	
NM_016631	34106209	34144169	GCFC1	deleted	*Gcfc1*	No		
NR_024622	34144410	34170016	C21orf49	deleted	Not found	Not found		
NM_001162496	34162983	34186053	C21orf62	deleted	*4932438H23Rik*	No		
NM_005806	34398238	34401500	OLIG2	deleted	*Olig2*	Yes	Homozygous mutation of this gene results in neonatal lethality, impaired development of motoneurons and oligodendrocytes, aphagia, hypotonia, abnormal posture and breathing.	
NM_138983	34442449	34444728	OLIG1	deleted	*Olig1*	Yes	Homozygous mutation of this gene results in impaired maturation of oligodendrocytes.	
NR_024102	34537775	34542541	C21orf54	deleted	Not found	Not found		
NM_207585	34602230	34636820	IFNAR2	deleted	*Ifnar2*	Yes	Mice with mutations of this gene have defects in immune responses involving, variously, NK cells, CD4+ and CD8+ T cells and B cells in response to induced and transplanted tumors, viruses, and double stranded DNA. These defects include diminished secretion of type I and type II interferons.	Yes
NM_000628	34638671	34669520	IL10RB	deleted	*Il10rb*	Yes	Most mice homozygous for a ko allele develop moderate to severe colitis without small intestinal involvement and splenomegaly with a hyperproliferative splenic red pulp.	Yes
NM_000629	34697213	34732128	IFNAR1	deleted	*Ifnar1*	Yes	Homozygotes for targeted null mutations exhibit increased susceptibility to viral infection, elevated levels of myeloid lineage cells in the peripheral blood and bone marrow, and reduced immune response to immunostimulatory DNA.	Yes
NM_005534	34775201	34809828	IFNGR2	deleted	*Ifngr2*	Yes	Mice homozygous for disruptions in this gene develop normally. Immune, hematopoietic defects. Diabetes?	Yes
NM_006134	34821447	34852281	TMEM50B	deleted	*Tmem50b*	No		
NM_001040192	34860237	34864023	DNAJC28	deleted	*Dnajc28*	No		
NM_001136006	34876237	34915198	GART	deleted	*Gart*	No		
NM_138927	34915349	34949812	SON	deleted	*Son*	No		
NM_017613	34950210	34961014	DONSON	deleted	*Donson*	No		
NM_145858	34961647	35014160	CRYZL1	deleted	*Cryzl1*	No		
NM_003024	35014783	35261609	ITSN1	deleted	*Itsn1*	Yes	Homozygous for a gene trapped allele exhibit embryonic lethal. Mice homozygous for a null allele exhibit some postnatal lethality and impaired vesicle recycling in surviving mice.	
NM_001697	35275756	35288158	ATP5O	deleted	*Atp5o*	No		
NM_032476	35445822	35515334	MRPS6	deleted	*Mrps6*	No		
NM_006933	35445869	35478561	SLC5A3	deleted	*Slc5a3*	Yes	Homozygous mutation of this gene results in lethality shortly after birth due to respiratory failure and abnormal development of peripheral nerves.	
NR_027266	35552977	35562220	C21orf82/NCRNA00310	deleted	Not found	Not found		
NM_172201	35736322	35743440	KCNE2	deleted	*Kcne2*	Yes	Digestive/alimentary and homeostasis defects. Gastric hyperplasia and abnormal parietal cell morphology.	
NM_058182	35747748	35761452	FAM165B	deleted	*Fam165b*	No		
NM_000219	35818987	35883613	KCNE1	deleted	*Kcne1*	Yes	Homozygotes for targeted and spontaneous null mutations exhibit head-shaking, circling, ataxia, and severe deafness associated with inner ear defects. Older mutants show increased numbers of T cells. Study of cardiac myocytes in one line showed physiologic defects.	Yes
NM_203418	35888783	35899261	RCAN1	deleted	*Rcan1*	Yes	Unstressed homozygous mutant mice show no overt phenotype other than a slight reduction in heart size and an impaired T helper 1 response. Stress-induced cardiac hypertrophy, however, is attenuated in mutant mice.	Yes
NM_053277	36041687	36090519	CLIC6	deleted	*Clic6*	No		
NR_024351	36096104	36109479	NCRNA00160	deleted	Not found	Not found		
NM_001754	36160097	36421595	RUNX1	partially deleted	*Runx1*	Yes	Mutations affect hematopoiesis, and in some cases result in defective angiogenesis and intraventricular hemorrhage. Null homozygotes die embryonically; heterozygotes have reduced erythroid and myeloid progenitor numbers. Skeletal defects.	Yes

A third mouse model for Down syndrome has recently been reported [39] in which Hsa21-syntenic regions on mouse chromosome 10, 16 & 17 (Mmu10, Mmu16 and Mmu17) are duplicated. Here all the mouse orthologs of Hsa21 genes are triplicated with a resulting neurological defect related to Down syndrome; impaired cognitive behaviour, reduced hippocampal LTP and hydrocephalus. Of all the mouse models available, this is perhaps the most similar to the human condition. However, no studies of auditory function have been reported for this mouse model and this could form the basis of a future hearing study.

## Materials and Methods

### Ethics Statement

All animal studies were licensed by the U.K. Home Office under the Animals (Scientific Procedures) Act 1986 and were approved by the University of Sheffield Ethical Review Committee (Home Office license PPL40/3278) and Wellcome Trust Sanger Institute (Home Office license PPL 80/2163).

### Mice

Tc1 mice carrying a copy of human chromosome 21 (HSA 21) were maintained on a mixed C57BL/6J×129S8 background [5].

### Auditory Brainstem Response (ABR) recordings

Ten Tc1 and 11 littermate controls were anaesthetised using urethane (ip) and placed on a heating blanket inside a sound attenuating booth. Sub-cutaneous needle electrodes were inserted in the skin on the vertex (active) and overlying the ventral region of the left (reference) and right (ground) bullae to record responses of the left ear. Stimuli were presented as free-field sounds from a speaker (Tucker Davis Technologies, FF1) whose leading edge was 10 cm in front of the mouse's interaural axis at an elevation of 30°. The sound delivery system was calibrated using an ACO Pacific 7017 microphone. For threshold determination, custom software and Tucker Davis Technologies hardware were used to deliver click (0.01 ms duration) and tone pip (3, 6, 18, 24, 30, 36 and 42 kHz of 5 ms duration, 1 ms rise/fall time) stimuli at a range of intensity levels from 10–100+ dB SPL in 3 dB steps. Averaged responses to 256 stimuli, presented at 42.2/s, were analysed and thresholds established as the lowest sound intensity giving a visually-detectable ABR response. For clicks, responses were also recorded from the right ear.

### Middle ear analysis

Following ABR, mice were killed by cervical dislocation and the right external and middle ears were examined in detail using a dissecting microscope for any signs of malformation or inflammation, including: appearance of excess cerumen in the external ear canal; thickening, whitening, sponginess or excess vascularisation of the bulla wall; clarity and vascularisation of the tympanic membrane; presence of fluid or white inflammatory material in the middle ear cavity; and solid masses or bony outgrowths of the middle ear wall or ossicles. In addition, following ABR the left side of the head of four Tc1 and four wildtype littermate controls were embedded in paraffin wax, serial sectioned at 8 µm, stained with haematoxylin and eosin and examined.

### Inner ear analysis

The right inner ear was dissected out, fixed in Bodian's fixative (75% ethanol, 5% formalin, 5% glacial acetic acid) for 2 hours, washed in distilled water for 30 mins before being placed in 70% ethanol for 24 hours. Inner ears were left in several changes of 3% KOH over 5 days to dissolve remaining soft tissues, and the bony structures of the ear were cleared by immersion in GEB (2∶2∶1 ratio of glycerol, 70% ethanol and benzyl alcohol) and thereafter, stored in a 1∶1 solution of glycerol and 70% ethanol for further clearing and examination using a dissecting microscope.

### Tissue preparation for single-hair cell electrophysiology

Apical-coil inner hair cells (IHCs: *n* = 14) and outer hair cells (OHCs: *n* = 12) from Tc1 mice [5] and their littermate controls were studied in acutely dissected organs of Corti from postnatal day 13–14 (P13–P14) for OHCs and P25–P26 for IHCs, where the day of birth is P0. Cochleae were prepared as previously described [21] in normal extracellular solution (mM): 135 NaCl, 5.8 KCl, 1.3 CaCl_2_, 0.9 MgCl_2_, 0.7 NaH_2_PO_4_, 5.6 d-glucose, 10 Hepes–NaOH, 2 Sodium pyruvate (pH 7.5, osmolality ∼308 mmol kg^−1^). Amino acids and vitamins (Eagle's MEM) were added from concentrates (Invitrogen, UK). Apical coils were transferred into a microscope chamber containing extracellular solution, and immobilized with a nylon mesh fixed to a stainless steel ring. The chamber (volume 2 ml) was perfused at a flow rate of about 10 ml·h^−1^ from a peristaltic pump and mounted on the stage of an upright microscope (Olympus, Japan). The organs of Corti were observed with Nomarski differential interference contrast optics (×60 water immersion objectives). Current and voltage recordings were made by exposing the basolateral surface of the cells using a suction pipette (tip diameter about 4 µm), which was filled with extracellular solution. Tissue samples from all mice were kept for genotyping. All animals were genotyped as previously described [5].

### Single-hair cell electrophysiology

IHCs and OHCs were whole cell patch clamped near body temperature (34–37°C) using an Optopatch amplifier (Cairn Research Ltd, Faversham, UK). Patch pipettes were pulled from soda glass capillaries (Harvard Apparatus Ltd., Edenbridge, UK; electrode resistances: 2–3 MΩ) and in order to reduce the electrode resistance coated with surf wax (Mr Zoggs SexWax, Ca, USA). The pipette filling solution contained (in mM): 131 KCl, 3 MgCl_2_, 1 EGTA-KOH, 5 Na_2_ATP, 5 Hepes-KOH, 10 Na_2_-phosphocreatine (pH 7.3, 294 mmol kg^−1^). Data were acquired using pClamp software and Digidata 1320A (Molecular Devices, USA), filtered at 2.5 kHz (8-pole Bessel), sampled at 5 kHz and stored on computer for off-line analysis (Origin: OriginLab, USA). Current recordings were corrected off-line for linear leakage. Membrane potentials were corrected for residual series resistance (*R*_s_) after compensation (1.6±1.1 MΩ, *n* = 26) and for a liquid junction potential (LJP) of −4 mV. The holding currents were plotted as zero current.

The large conductance Ca^2+^ activated K^+^ current *I*_K,f_ was isolated by measuring its amplitude at the membrane potential of −25 mV and at 2 ms from the start of the voltage step [16]. The negatively activating *I*_K,n_ was measured in isolation as the deactivating tail currents (difference between instantaneous and steady-state inward currents) for voltage steps from the holding potential to −124 mV [15].

Statistical comparisons of means were made by the two-tailed *t*-test. Two-way ANOVA, followed by the Bonferroni test, was used to compare voltage responses from control and Tc1 hair cells. Mean values are quoted ± SEM where p<0.05 indicates statistical significance. In some of the figures statistical significance is indicated by asterisks.
